# Statistical analysis plan for the WOMAN-ETAPlaT study: Effect of tranexamic acid on platelet function and thrombin generation

**DOI:** 10.12688/wellcomeopenres.10105.2

**Published:** 2017-06-14

**Authors:** Kastriot Dallaku, Haleema Shakur, Phil Edwards, Danielle Beaumont, Ian Roberts, Sumaya Huque, Maria Delius, Ulrich Mansmann

**Affiliations:** 1 Institute for Medical Information Sciences, Biometry and Epidemiology, Klinikum Großhadern, Ludwig-Maximilian University, Munich, Germany; 2 University Hospital of Obstetrics Gynaecology “Koco Gliozheni, Tirana, Albania; 3 Clinical Trials Unit, London School of Hygiene & Tropical Medicine, London, UK; 4 Department of Obstetrics and Gynaecology, Ludwig Maximilian University of Munich, Munich, Germany

**Keywords:** Antifibrinolytic, Multiplate Analyser, Thrombin Generation Assay, Statistical Analysis Plan

## Abstract

**Background**. Postpartum haemorrhage (PPH) is a potentially life-threatening complication for women, and the leading cause of maternal mortality. Tranexamic acid (TXA) is an antifibrinolytic used worldwide to treat uterine haemorrhage and to reduce blood loss in general surgery. TXA may have effects on thrombin generation, platelet function and coagulation factors as a result of its inhibition on the plasmin.

**Methods**. WOMAN ETAPlaT is a sub-study of the World Maternal Antifibrinolitic trial (WOMAN trial). All adult women clinically diagnosed with PPH after a vaginal delivery or caesarean section, are eligible for inclusion in the study. Blood samples will be collected at the baseline and 30 minutes after the first dose of study treatment is given. Platelet function will be evaluated in whole blood immediately after sampling with Multiplate® tests (ADPtest and TRAPtest). Thrombin generation, fibrinogen, D-dimer, and coagulation factors vW, V and VIII will be analysed using platelet poor plasma.

**Results.** Recruitment to WOMAN ETAPlaT started on 04 November 2013 and closed on 13 January 2015, during this time  188 patients were recruited. The final participant follow-up was completed on 04 March 2015. This article introduces the statistical analysis plan for the study, without reference to unblinded data.

**Conclusion. **The data from this study will provide evidence for the effect of TXA on thrombin generation, platelet function and coagulation factors in women with PPH.

**Trial registration**: ClinicalTrials.gov Identifier: NCT00872469; ISRCTN76912190

## Abbreviations

ADPtest: Test of Adenosine Di Phosphate (ADP); AUC: Area Under Curve; CI: confidence Interval; CONSORT: Consolidated Standards of Reporting Trials; ETP: Endogenous Thrombin Potential; FAS: Full Analysis Set; FV: Coagulation Factor V; FVIII: Coagulation Factor VIII; CBC: Complete Blood Count; IBE: Institute for Medical Information Sciences, Biometry and Epidemiology; Hb: Hemoglobin; Ht: Hematocrit; LT: Lag Time; PtH: Peak to Height; MPV: Mean Platelet Volume Mean Platelet Volume; PP: Per-Protocol; R: software package R (r-project.org); SAP: Statistical Analysis Plan; SD: Standard Deviations; SOP: Standard Operating Procedure; TGA: Thrombin Generation Assay; TXA: Tranexamic Acid; TRAPtest: Test of Thrombin Receptor of Thrombocyte; vWF: Coagulation Factor Von Willebrand.

### Preface

The purpose of the statistical analysis plan (SAP) is to ensure the credibility of the study findings by pre-specifying the statistical approaches to the analysis of the study data prior to hard locking the database and unblinding of the WOMAN ETAPlaT trial data. To prevent outcome bias and selective reporting, a detailed SAP is presented in order to avoid post hoc decisions that may influence the interpretation of the results and the statistical analyses of the final data.

This SAP is a technical extension of the WOMAN ETAPlaT study protocol (Version 1.1, dated August 29, 2014), which is published elsewhere ( Dallaku *et al*., 2016). The SAP follows the principles of the International Conference on Harmonization (ICH) guidelines E3, E6 and E9 ( ICH, 2016).

## Study objectives and endpoints of WOMAN-ETAPlaT

### Objectives of the study

Primary objectives: This study will assess if TXA, by inhibiting the plasmin, has an effect on decrease over time on the continuous response variable, thrombin generation assay (TGA) parameter: endogenous thrombin potential (ETP). Secondary objectives: evaluation of the effect of TXA on women with PPH, through monitoring the platelet function (multiplate analyser), plasmatic levels of fibrinogen, D-dimer, coagulation factor V (FV), coagulation factor VIII (FVIII), coagulation factor von Willebrandt (vWF) and other TGA parameters: lag time (LT), time to peak (TtP), and peak height (Ph).

### Endpoints

***Primary efficacy endpoint.* The primary efficacy endpoint** is the TGA parameter – ETP (nM/minute). Values of ETP will be measured in venous blood samples. Blood samples will be collected at the baseline and at 30 ±15 minutes after the first dose of study treatment is given. Analysis will be performed on processed, separated platelet poor plasma, and preserved in deep freeze, as described in the ETAPlaT protocol ( Dallaku *et al*., 2016).

***Secondary efficacy endpoints.***Secondary outcomes will include parameters measuring the effect of TXA on other TGA parameters (LT, TtP, and Ph - we will transform variables prior to analysis if there is evidence that distributions are skewed), fibrinogen, D-dimer and coagulation factors V, VIII, vWF (measured on processed plasma samples as described on primary endpoint) and platelet function analysed by Multiplate ^®^ tests (ADPTest, TRAPTest – measured on whole blood). 

## Study methods

### Study design

The **WOMAN-ETAPlaT** is a sub-study of the WOMAN trial, an international randomised, double blinded, placebo-controlled trial. The ETAPlaT sub-study has the same study design as the WOMAN trial, but includes some additional laboratory tests. Women who fulfil the eligibility criteria for the WOMAN trial, including adult women clinically diagnosed with primary PPH after a vaginal delivery (>500 ml blood loss), a caesarean delivery (>1000 mL blood loss), or enough blood loss to compromise the haemodynamic status will be randomized. Women with PPH for whom the physician believes there is an indication or contraindication to use TXA, will be excluded.

Immediately after randomization, women will receive the trial treatment by i/v administration of either TXA (1 gram) or placebo (NaCl solution 0.9%). If after 30 minutes the haemorrhage continues or if it stops and restarts within 24 hours after randomization, a second dose may be given. This second dose is not relevant for the current sub-study.

### Randomisation, blinding and ethics approval

Packs with study drugs were prepared according to the randomization list, generated by an independent statistician. The active study drug (TXA) and placebo (NaCl 0.9%) will be identical in appearance, and randomization number will be used for identification of packs. Each box has eight packs of study drugs, and the lowest number pack will be used first. After completing the consent procedure, eligible patients are randomized to receive either TXA or placebo. Both the patients and the medical representatives participating in the study are masked to the treatment distribution. Ethical approval was obtained in July 11, 2013 by National Ethics Committee in Tirana, Albania (ref. 62 and 81) and in October 28, 2013 by London School of Hygiene and Tropical Medicine, UK (ref. 6518).

### Study variables and study schema

Details concerning the baseline (treatment) and follow-up (post treatment) period together with the frequency and timing of relevant variables or assessments are displayed at Table 1.

**Table 1.** Datasets that will be generated from the study and will be included in a database.

Variables	Time period
MultiplateAnalyser test – ADPtest (.csv file)	Baseline data and 30 ± 15 min after study treatment
MultiplateAnalyser test – TRAPtest (.csv file)	Baseline data and 30 ± 15 min after study treatment
Thrombin generation assay (.csv file)	Baseline data and 30 ± 15 min after study treatment
Coagulation factors vW, V, VIII, Fib, and DDim (.csv file)	Baseline data and 30 ± 15 min after study treatment
Clinical data collection forms (.csv file)	Baseline data and follow-up
Full blood count (.csv file)	Baseline data and within 12 hrs after randomization

## Sample size

The sample size calculation is focused on the first component of the primary endpoint: change over time of ETP in women with PPH. In a previous study ( McLean *et al.,* 2012) ETP in patients with term pregnancy, within each subject group, was normally distributed with a mean of 2410 nM/min and standard deviation (SD) of 543 nM/min.

Assuming a correlation of 0.6 between two time points, the standard deviation of the change can be calculated as 485 {sqrt[2×543^2×(1-0.6)])}. We assume a decrease in ETP of 10% (243 nM/min) over time in the TXA group and no change in the placebo group. To detect an ETP difference of 243 nM/min between groups at a 5% significance level with a power of 80%, two groups each with 88 patients are needed.

A correlation of 0.6 is seen as a conservative estimate as the within individual changes in TGA over time is strictly controlled, which implies a high correlation between time points.

## General considerations

### Timing of analyses

Participant recruitment started on 4 November 2013 and was completed on 13 January 2015. The final participant follow-up was completed on 04 March 2015.

All final analyses will be performed on the database at LSHTM (London, UK) which consists of several tables ( Table 1). After documenting all data collection forms (DCF) data, data cleaning and query resolution, the following prerequisites for unblinding must be fulfilled: the resolution of all queries concerning DCF and laboratory results, and the finalisation of the SAP document.

All these processes before database locking must take place to comply with requirements.

Following data integrity checks the database will be locked after October 2016 and the statistical analysis specified in the SAP and approved by the Trial Steering Committee (TSC), will be performed in advance of the WOMAN trial database lock. In the event the TSC approves analysis before the end of the WOMAN trial, only the independent Data Monitoring Committee (DMC) statistician will be aware of each participant’s treatment allocation. Each participant will be allocated a unique identifier (different from that of the WOMAN trial before unblinding). This is to ensure that the WOMAN trial blinding is not compromised in any way.

### Analysis populations

**Figure 1.**
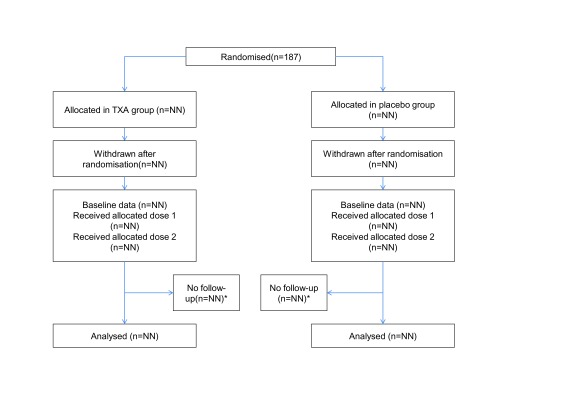
ETAPlaT sub-study flowchart:* Patients for whom there is no information on the primary endpoint.

***Full analysis set (FAS).*** The primary efficacy analysis follows the principle of intention to treat (ITT), which implies that study data are analysed based on the original allocation of patients, regardless of a treatment received. Withdrawals, participants lost to follow-up and participants who did not adhere fully to the WOMAN ETAPlaT study protocol, will not be excluded from the primary efficacy analyses provided that they satisfy major entry criteria. Women who have withdrawn consent to use their data will be excluded from the ITT analysis. Women with a follow-up measure but no baseline measure will be included in the ITT analysis ( Figure 1).

***Per-protocol set (PP).*** The PP set consists of all patients who did not substantially deviate from the WOMAN ETAPlaT protocol as to be determined on a per-subject basis. The PP set of subjects defining a subset of the FAS is characterised as follows: (1) with measurements of outcome available, (2) no major protocol violations are detected: (a) Failure to administer the study drug (first dose), (b) Failure to collect blood sample (baseline or follow-up), (c) Exceeding time-frame of 30 min ± 15 minutes between the study drug administration and follow-up blood sample collection.

### Covariates and subgroups

There exists no *a priori* hypothesis of subgroup differences regarding treatment effects. Hence, no pre-planned confirmatory subgroup analyses will be performed to explore evidence for a difference in treatment effects (interaction effect).

## Summary of study data

All continuous variables will be summarised by treatment group (TXA/placebo) using the following descriptive statistics: sample size (N), mean ±standard deviation (SD), median, maximum and minimum. The absolute frequency and percentages of observed levels will be reported for all categorical variables. Summary statistics will be displayed overall and stratified by the treatment group. The key baseline characteristics selected are presented in Table 2.

**Table 2.** Baseline characteristics of participants before randomization.

	TXA (n, %)	Placebo (n, %)
Patient height, weight, BMI		
Parity: nullipara (0), multipara (>1)		
Gestational age at birth: <37 or ≥37 weeks		
Any concomitant diseases of pregnancy: Preclampsia, Chorioamnionitis, Diabetes, Placental abruption, Placenta previa, Previous PPH		
Use of anaesthesia: General or spinal or none		
Duration of labour of vaginal births (min) 1 ^st^, 2 ^nd^ and 3 ^rd^ stage of labour		
Fetal birth weight: < 4000 gr or ≥ 4000 gr		
Hb level at entry of the study		
Platelet count at entry of the study		
Fibrinogen level at entry of the study		
Amount of blood loss		
Primary cause of PPH Atony, placenta, trauma, other		
Mode of delivery:vaginal- caesarean		
Additional doses of uterotonics		

### Baseline characteristics

In a study about the mode of anaesthesia related to PPH after caesarean deliveries, a significantly higher amount of blood loss during general anaesthesia compared to spinal anaesthesia was previously reported ( Aksoy *et al.,* 2015). The reduction of blood loss in neuroaxial anaesthesia maybe was caused partly from hypotension and by fluids infused in these women ( Heesen *et al.*, 2013). Another role in increased blood loss during general anaesthesia was reported from volatile anaesthetics which may decrease the myometrium contractility ( Yoo *et al.*, 2006) and as result can cause uterine atony. Also, volatile anaesthetics with their inhibition effect on platelet aggregation ( Yuki *et al.,* 2013) can increase the risk for PPH.

Dionne *et al.* (2015) mentioned that prolonged second stage labour was associated with increased risk and severity of PPH. Other potential risk factors for PPH were null-parity, high birth-weight and antepartum anaemia, where low levels of haemoglobin before delivery were associated with a low efficacy of haemostasis mechanism promoted by red cells and platelets ( Biguzzi *et al.*, 2012), and as result with an increased risk for PPH.

## Efficacy analyses of primary outcome

Both groups of randomised patients, those allocated to the TXA group and placebo group will be compared and analysed on an intention-to-treat basis, irrespective of treatment given. The results will be presented as appropriate effect estimates with a measure of precision (95% CI). In analysis of the primary outcome(TGA– ETP), the TXA group will be compared with the placebo group, based on analysis of covariance (ANCOVA) which adjusts for the baseline measurement of the respective outcome. In a sensitivity analysis we also add an adjustment for the length of time between two measurements (30±15 minutes) of the baseline and follow-up TGA - ETP value ( Table 3).

**Table 3.** Evaluation of TXA effect compared to placebo on TGA –ETP, in women with PPH.

	TXA (n)			Placebo (n)			95% CI p-value		
**Primary outcome**	Before	After*	Delta	Before	After*	Delta	
**TGA – ETP** (nM*min)						

* 30 minutes

## Efficacy analyses of secondary outcomes

The same analyses as for primary outcome will be performed for the blood parameters which are secondary endpoint parameters of Multiplate ^©^ tests (ADPtest and TRAPtest), fibrinogen, D-dimer, coagulation factors V, VIII, vWF and other TGA parameters (lag time, time to peak, peak height) ( Table 4). The results will be presented as appropriate effect estimates with a measure of precision (95% CI).

**Table 4.** Effect of tranexamic acid on coagulation factors and platelet function.

		TXA (n)			Placebo (n)			Difference 95% CIP-value
**TGA parameters**		Before	After*	Δ	Before	After*	Δ	
	Lag time(min)							
	Time to peak(min)							
	Peak height (nM)							
**Multiplate tests**								
	ADPtest(AU*min)							
	TRAPtest(AU*min)							
**Coagulation tests**								
	FV (%)							
	FVIII (%)							
	vWF (%)							
	D-dimer (mg/L)							
	Fibrinogen (mg/dL)							

* 30 minutes

All statistical analyses will be conducted with the statistics package R and STATA.

### Influence of pregnancy diseases and PPH causes on the TXA effect for study outcomes

In pregnant women diagnosed with preeclampsia, preterm labour and chorioamnionitis, was found an increased thrombin generation ( Mastrolia *et al.,* 2014). Chaiworapongsa *et al.* (2002) also reported increased thrombin generation in patients diagnosed with preeclampsia and fetal growth restriction. Macey *et al*. (2010) comparing normal pregnancies with and without preeclampsia, reported an increased thrombin formation and increased platelet activation. Increased thrombin generation and platelet activity was found also in women with a history of preeclampsia ( Rafik Hamad *et al.,* 2009).

There is evidence that the haemostatic impairment that occurs in haemorrhage during pregnancy and delivery is different from trauma-induced haemorrhage. The type and rate of onset of coagulopathies differ depending on the main cause of obstetric haemorrhage ( Collis & Collins, 2015). Regarding haemostasis changes during obstetric haemorrhage, there is little data. The type, severity and rate of onset of the coagulopathy vary with the aetiology of bleeding.

Allard *et al.* (2014) raised the question, whether women with postpartum haemorrhage exhibit different changes from other patients who have haemorrhage during general surgery or trauma? Some of the underlying causes of postpartum haemorrhage such as uterine atony and genital tract trauma were often associated with no significant coagulopathy ( Collins *et al*., 2014). But, when the cause of PPH was placental abruption, it may be associated with rapid consumptive coagulopathy characterised with clinically severe haemostatic impairment ( Thachil & Toh, 2009).

### Influence of PPH severity, fibrinogen and platelet count on the TXA effect for study outcomes

Low plasmatic level of fibrinogen such as 2 – 3 g/L and especially less than 2 g/L at the beginning or on-going of PPH, was associated with an increased amount of blood loss, increased need for blood transfusion and requirements for invasive procedures. Low platelet number at the diagnosis of PPH was associated with the requirement for invasive procedures ( Collins *et al*., 2014). Simon *et al.* (1997), observed an increased frequency of PPH, when in early labour platelet count is <100000/mm³ or fibrinogen level is <2.9mg/L or both. A study has reported that thrombocytopenia <80 000/mm³ was associated with an increased incidence of severe PPH ( Dikman *et al*., 2014), however, another study did not found any association between thrombocytopenia and severe PPH ( Jones *et al.,* 2016).

A systematic review ( Simonazzi *et al.*, 2016) confirmed that TXA can significantly decrease the incidence of PPH with an amount of blood loss >500 ml and severe PPH with blood loss > 1000ml. Consume of coagulation factors and platelets were reported in cases with severe postpartum bleeding. The decrease of fibrinogen level, antithrombin activity, and the platelet count was observed in PPH cases with blood loss >2000 ml.

**Figure 2.**
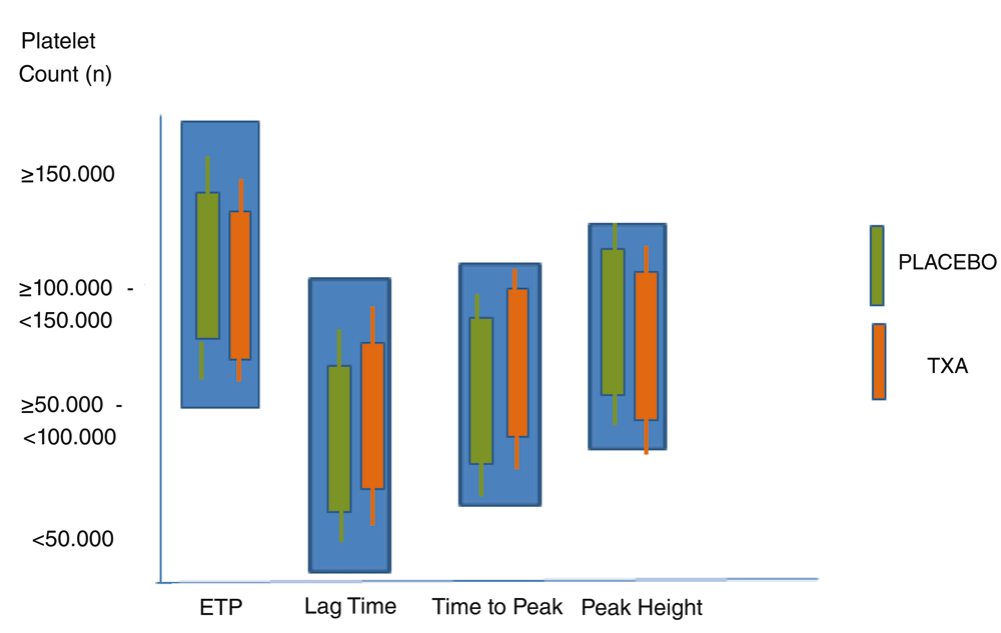
Effect of TXA on TGA parameters, regarding the platelet count before randomization (graph model using arbitrary data).

A series of graphs will present each TGA parameter (ETP, LT, TtP, Ph) as a panel of 4 boxplots which show the measured values for the treatment groups split with respect to baseline platelet count categorized as follows: <50000, 50000–100000, 100000–150000, ≥ 150000 ( Figure 2). Similar graphs will display the coagulation factors (FV, FVIII, vWF, fibrinogen and D-dimer) and Multiplatetests (ADPtest and TRAPtest) measured values, with respect to platelet count for both treatment groups. Differences between the TXA/placebo groups will be tested using the non-parametric Kruskall-Wallis test.

Definition of boxplots: The publication will use the boxplot as implemented in the basic statistics package of R by the function “boxplot.stats”. The two ‘hinges’ are versions of the first and third quartile. They equal the quartiles for odd n (where n <- length(x)) and differ for even n. The notches extend to +/-1.58 IQR/sqrt(n). See in Chambers *et al.* (1983, p. 62), given in McGill *et al.* (1978, p. 16).

**Figure 3.**
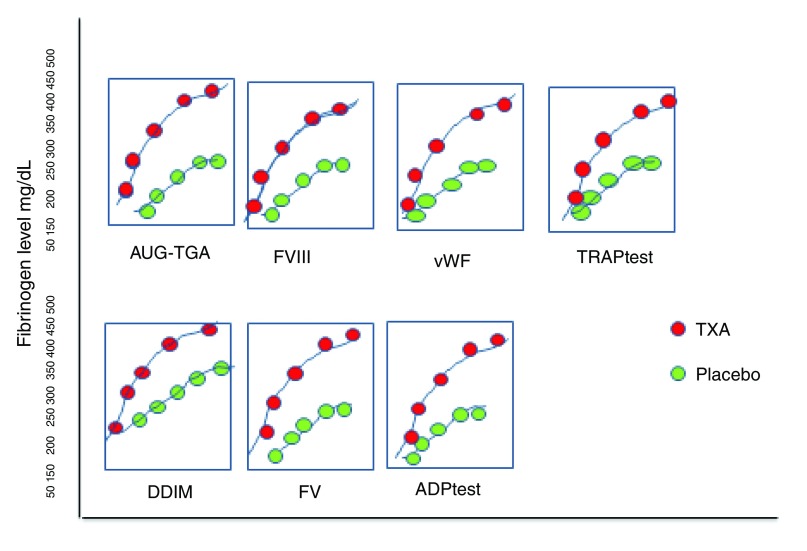
Effect of TXA on coagulation factors and platelet function, regarding the baseline fibrinogen level (graph model using arbitrary data).

Baseline fibrinogen level and a panel of 7 dot-plots showing the corresponding individual measurements for each individual patient with respect to TGA-ETP, FV, FVIII, vWF, D-dimer, ADPtest and TRAPtest for both treatment groups TXA/placebo ( Figure 3).

The dots will be color-coded with respect to the treatment groups, a smoother will be presented in the corresponding colour, the Spearman correlation will be calculated and a 95% CI for each treatment group will be presented. Also, the difference between the correlation coefficients of the treatment groups will be calculated and a 95% confidence interval will be presented. The confidence intervals related to the Spearman correlation coefficient will be calculated by bootstraping.

Two panels of graphs will present the ADPtest parameters as well as the TRAPtest parameters. They will show individual measurements for aggregation, velocity, and AUC of TXA/placebo groups correlated with individual platelet count. These graphs (similar to Figure 2) will be produced for baseline, after treatment and change values.

### Per-protocol analyses

All analyses as described in of the section ‘Efficacy analysis for primary and secondary outcomes’ will be repeated using the PP analysis set.

## Safety

For all patients randomized in the ETAPlaT study who received TXA or placebo, from the moment of randomization and until 42 days later, any treatment safety data is available. Adverse events (AE) and serious adverse events (SAE), classified on the basis of MedDRA terminology ( ICH, 2016), will be reported to an independent data monitoring committee (DMC), which is already in place for the WOMAN trial. All information regarding the AEs, SAEs will be registered for each patient affected, by date of onset and resolution, severity, frequency and outcome of AEs. The statistics will be organized by comparing TXA/placebo groups of patients with at least one adverse event.

## Adherence

We report measures of compliance by trial arm: number of damaged or misallocated packs, time from randomisation to drug administration, time from baseline to 2nd measurement, number of 2nd doses administered.

## Conclusions

This article presents the principles of statistical analysis of the WOMAN ETAPlaT study, in order to ensure the credibility of the study findings by pre-specifying the statistical approaches to the analysis of the study data. The detailed SAP presented will help to prevent outcome bias, selective reporting and to avoid post hoc decisions that may influence the interpretation of the results and the statistical analyses of the final data. The data from this study will provide evidence for the effect of TXA on thrombin generation, platelet function and coagulation factors in women with PPH.

## References

[ref-1] Aksoy H Aksoy Ü Yücel B: Blood loss in elective cesarean section: is there a difference related to the type of anesthesia? A randomized prospective study. *J Turk Ger Gynecol Assoc.* 2015;16(3):158–63. 10.5152/jtgga.2015.15034 26401109 PMC4560473

[ref-2] Allard S Green L Hunt BJ: How we manage the haematological aspects of major obstetric haemorrhage. *Br J Haematol.* 2014;164(2):177–188. 10.1111/bjh.12605 24383841

[ref-3] Biguzzi E Franchi F Ambrogi F: Risk factors for postpartum hemorrhage in a cohort of 6011 Italian women. *Thromb Res.* 2012;129(4):e1–e7. 10.1016/j.thromres.2011.09.010 22018996

[ref-4] Chaiworapongsa T Yoshimatsu J Espinoza J: Evidence of *in vivo* generation of thrombin in patients with small-for-gestational-age fetuses and pre-eclampsia. *J Matern Fetal Neonatal Med.* 2002;11(6):362–367. 10.1080/jmf.11.6.362.367 12389649

[ref-25] Chambers JM Cleveland WS Kleiner B: Graphical Methods for Data Analysis.1983;62 Reference Source

[ref-5] Collins PW Lilley G Bruynseels D: Fibrin-based clot formation as an early and rapid biomarker for progression of postpartum hemorrhage: a prospective study. *Blood.* 2014;124(11):1727–36. 10.1182/blood-2014-04-567891 25024304

[ref-6] Collis RE Collins PW: Haemostatic management of obstetric haemorrhage. *Anaesthesia.* 2015;70(Suppl 1):78–86,e27–8. 10.1111/anae.12913 25440400

[ref-7] Dallaku: Effects of tranexamic acid on platelet function and thrombin generation (ETAPlaT): WOMAN trial sub-study. *F1000Res.* 2016. 10.12688/wellcomeopenres.9964.1 PMC5234699 28090594

[ref-8] Dikman D Elstein D Levi GS: Effect of thrombocytopenia on mode of analgesia/anesthesia and maternal and neonatal outcomes. *J Matern Fetal Neonatal Med.* 2014;27(6):597–602. 10.3109/14767058.2013.836483 23962227

[ref-9] Dionne MD Deneux-Tharaux C Dupont C: Duration of Expulsive Efforts and Risk of Postpartum Hemorrhage in Nulliparous Women: A Population-Based Study. *PLoS One.* 2015;10(11): e0142171. 10.1371/journal.pone.0142171 26555447 PMC4640501

[ref-11] Heesen M Hofmann T Klöhr S: Is general anaesthesia for caesarean section associated with postpartum haemorrhage? Systematic review and meta-analysis. *Acta Anaesthesiol Scand.* 2013;57(9):1092–1102. 10.1111/aas.12178 24003971

[ref-12] ICH guidelines. Accessed online on October; 2016. Reference Source

[ref-13] Jones RM de Lloyd L Kealaher EJ: Platelet count and transfusion requirements during moderate or severe postpartum haemorrhage. *Anaesthesia.* 2016;71(6):648–656. 10.1111/anae.13448 27062151

[ref-14] Macey MG Bevan S Alam S: Platelet activation and endogenous thrombin potential in pre-eclampsia. *Thromb Res.* 2010;125(3):e76–e81. 10.1016/j.thromres.2009.09.013 19822350

[ref-15] Mastrolia SA Mazor M Loverro G: Placental vascular pathology and increased thrombin generation as mechanisms of disease in obstetrical syndromes. *Peer J.* 2014;2:e653. 10.7717/peerj.653 25426334 PMC4243334

[ref-16] McGill R Tukey JW Larsen WA: Variations of box plots. *Am Stat.* 1978;32(1):12–16. 10.2307/2683468

[ref-17] McLean KC Bernstein IM Brummel-Ziedins KE: Tissue factor-dependent thrombin generation across pregnancy. * Am J Obstet Gynecol.* 2012;207(2):135.e1–6. 10.1016/j.ajog.2012.05.027 22840726 PMC3661010

[ref-19] Rafik Hamad R Curvers J Berntorp E: Increased thrombin generation in women with a history of preeclampsia. *Thromb Res.* 2009;123(4):580–586. 10.1016/j.thromres.2008.03.022 18501408

[ref-20] Simon L Santi TM Sacquin P: Pre-anaesthetic assessment of coagulation abnormalities in obstetric patients: usefulness, timing and clinical implications. *Br J Anaesth.* 1997;78(6):678–683. 10.1093/bja/78.6.678 9215019

[ref-21] Simonazzi G Bisulli M Saccone G: Tranexamic acid for preventing postpartum blood loss after cesarean delivery: a systematic review and meta-analysis of randomized controlled trials. *Acta Obstet Gynecol Scand.* 2016;95(1):28–37. 10.1111/aogs.12798 26698831

[ref-22] Thachil J Toh CH: Disseminated intravascular coagulation in obstetric disorders and its acute haematological management. *Blood Rev.* 2009;23(4):167–76. 10.1016/j.blre.2009.04.002 19442424

[ref-23] Yoo KY Lee JC Yoon MH: The effects of volatile anesthetics on spontaneous contractility of isolated human pregnant uterine muscle: a comparison among sevoflurane, desflurane, isoflurane, and halothane. *Anesth Analg.* 2006;103(2):443–7, table of contents. 10.1213/01.ane.0000236785.17606.58 16861431

[ref-24] Yuki K Bu W Shimaoka M: Volatile anesthetics, not intravenous anesthetic propofol bind to and attenuate the activation of platelet receptor integrin αIIbβ3. *PLoS One.* 2013;8(4):e60415. 10.1371/journal.pone.0060415 23573252 PMC3616120

