# Supplementary material for: Statistical analysis plan for the WOMAN-ETAPlaT study: Effect of tranexamic acid on platelet function and thrombin generation
Source: Wellcome Open Res. 2017 Jun 14;1:30. Originally published 2016 Dec 15. [Version 2] doi: 10.12688/wellcomeopenres.10105.2 (PMC5390850; doi:10.12688/wellcomeopenres.10105.2)
Supplement: Peer review history

# Peer review: Referee response for version 2

**DOI:** 10.21956/wellcomeopenres.12743.r23487

**Published:** 2017-07-10

**Authors:** Manisha Nair, Louise Linsell

**Affiliations:** 1 National Perinatal Epidemiology Unit (NPEU), Nuffield Department of Population Health (NDPH), University of Oxford, Oxford, UK; 2 National Perinatal Epidemiology Unit, University of Oxford, Oxford, UK

Thank you for updating the article using our suggestions.

We have read this submission. We believe that we have an appropriate level of expertise to confirm that it is of an acceptable scientific standard.

# Peer review: Referee response for version 2

**DOI:** 10.21956/wellcomeopenres.12743.r23901

**Published:** 2017-06-29

**Authors:** Stephen J. Senn

**Affiliations:** 1 Competence Centre for Methodology and Statistics, Luxembourg Institute of Health, Strassen, Luxembourg

In view of the proposed amendment, I approve this plan.

I have read this submission. I believe that I have an appropriate level of expertise to confirm that it is of an acceptable scientific standard.

# Peer review: Referee response for version 1

**DOI:** 10.21956/wellcomeopenres.10886.r20856

**Published:** 2017-04-06

**Authors:** Manisha Nair, Louise Linsell

**Affiliations:** 1 National Perinatal Epidemiology Unit (NPEU), Nuffield Department of Population Health (NDPH), University of Oxford, Oxford, UK; 2 National Perinatal Epidemiology Unit, University of Oxford, Oxford, UK

This is an interesting study. The authors may want to consider the following points:

**Comments from Manisha Nair**

1. I understand that the primary aim of this study was to generate evidence about the effect of TXA on thrombin generation, platelet function and coagulation factors in women with PPH. However, this study could also provide answers to why TXA had or did not have the desired effect. In this context it would be important to analyse how the TXA effect is modified by baseline haemoglobin, platelets and coagulation factors, especially considering a high prevalence of anaemia during pregnancy in LMICs. I am surprised that the authors did not pre-specify this, but intend to conduct this only as an exploratory analysis. Clearly, these sub-group analyses will be underpowered to derive any meaningful results.
  In the second paragraph under sub-heading “Exploratory subgroup analyses” (page-6), authors write, “A further sensitivity analysis will be done by adjusting for severity of PPH (Table 5) and fibrinogen level or platelet count” – I presume this is fibrinogen or platelet count at baseline?
2. In Table 2, authors have grouped induction and augmentation of labour. These are two different interventions and would probably be better to report them separately.
3. Will there be any information about the management of third stage of labour for those who underwent vaginal delivery?
4. The participants will be recruited at a point when they are diagnosed with PPH, so some women particularly in LMICs are likely to be rushed into the hospital and may not have records of antenatal care. Will it be possible to have complete information on preclampsia, chorioamnionitis, diabetes, placental abruption, placenta praevia, previous PPH and other baseline parameters? Therefore, will it not be difficult to ascertain the cause of PPH at least for some women? The authors do not comment on these issues and resultant missing data and how this will be handled in the analyses.
5. My concern is that the authors propose a lot of tests with a small sample. Although these are important, I am not sure if the results will be meaningful. While we will know if TXA had an effect on three coagulation parameters, but we will not know in which population groups this drug works well and in which it may not.

**Comments from Louise Linsell**

**Page 3 Study design, 1st paragraph**

 Add that this sub-study is in a single centre.

 Last sentence doesn't make sense. Suggest, "Women with PPH for whom the physician believes there is an indication or contraindication to use TXA will be excluded."

**Page 4 Full analysis dataset, 1st paragraph**

 Please clarify that women who have withdrawn consent to use their data will be excluded from the ITT analysis. Will women with a follow-up measure but no baseline measure be included in the ITT analysis?

**Page 4 Per protocol set, 1st paragraph**

 The bullet points after "No major protocol violations were detected" are not clear and do not make sense.

 In addition to completion of blood samples add "with measurements of outcome available"

**Page 4 Covariates and subgroups**

 Please clarify all pre-specified subgroup analysis and methods of analysis, e.g. test for interaction and tie up with paragraph on **Page 6 Exploratory subgroup analyses** and the **last paragraph on page 7**. Provide detail on the coding of the sub group variables, and which outcomes will be included for which subgroup variables all in one place. Will subgroup analysis be conducted on the ITT set, PP set or both?

**Page 4 Summary of study data, 1st paragraph**

 Remove sentence, "If considered appropriate these data are summarised by centre as well." as there is only one centre.

**Page 5 Efficacy analyses of primary outcome**

 The last sentence refers to a subgroup analysis - this should go in the section detailing subgroup analyses.

**Page 5 Effect of TXA on thrombin generation, 1st and 2nd paragraph**

 Not sure why this text is included in the SAP at this point - it belongs either in the protocol or the background.

**Page 6 Efficacy analyses of secondary outcomes, 2nd paragraph**

 "The remaining secondary outcomes" - these are not listed in the protocol?

**Figure 1**

 Please provide labels or specify what the measurements in the boxplots represent, i.e. are the maximum, minimum values represented by the end of the line, or the end of the box?

**General comments**

**Subgroup and exploratory analyses**

 The text related to subgroup analyses is scattered and confusing. It is stated that these are exploratory, however if they are pre-specified and statistical tests are conducted, then the results should be reported in full as part of the main trial report. There are also blocks of text reporting evidence from other studies that detract attention and disrupt the flow of the SAP. Perhaps it would be clearer to have one SAP for the analysis and reporting of the main trial results and any pre-specified subgroup analysis in which all results are reported in full and a second SAP outlining any further exploratory analysis investigating the pathology/epidemiology.

**CONSORT Flowchart**

 Please include a CONSORT flowchart

**Adherence**

 It would be useful to report measures of compliance and other useful information by trial arm, e.g. number of damaged, misallocated packs, time from randomisation to drug administration, time from baseline to 2nd measurement, number of 2nd doses administered.

We have read this submission. We believe that we have an appropriate level of expertise to confirm that it is of an acceptable scientific standard, however we have significant reservations, as outlined above.

# Response

**Published:** 2017-05-29

**Authors:** Kastriot Dallaku

**Affiliations:** Hospital of Ludwig Maximilian University of Munich, Germany

We would like to thank you for helpful and constructive feedback.

Below you find our answers regarding comments of **Manisha Nair**:

1. We approach the search for factors at baseline which modify the effect of TXA on thrombin generation (etc.) by the formal analysis of an interaction between the treatment and the corresponding baseline parameter on the specific outcome. As mentioned in your comment, we did not power the study to detect specific interactions.
  Furthermore, we did not prespecify subgroup analyses to assess possible effect modifications of baseline parameters on the treatment.
  In the section “Covariates and Subgroups” we addressed this issue. According to your comments we tried to make this point more obvious.We see now the task to reduce the reporting of post hoc subgroup analyses.
2. Since we have grouped the patient with induced and augmentation of labour in data collection form, it is difficult to separate, so we will remove these data from the SAP. 
3. There are no data concerning management of third stage of labour.
4. There are no missing values regarding concomitant and preexisting diseases of pregnancy.
5. The total sample size is small and the subgroup analyses run the risk of false positive. We will react by removing the exploratory subgroup analysis. 

Below you find our answers regarding comments of **Louise Linsell**: 

1. Page 3 Study design, 1st paragraph: Many thanks for your comment. We changed the sentence in the SAP.
2. Page 4 Full analysis dataset, 1st paragraph:Thanks, we address this issue now in the revised SAP version. Missing values will be imputed.
3. Page 4 Per protocol set, 1st paragraph: We clarified this formulation in the revised SAP.
4. Page 4 Covariates and subgroups: The total sample size is small and the subgroup analyses run the risk of false positive. We removed the exploratory subgroup analysis. 
5. Page 4 Summary of study data, 1st paragraph: Done
6. Page 5 Efficacy analyses of primary outcome: Done
** **
7. Page 5 Effect of TXA on thrombin generation, 1st and 2nd paragraph: We removed the paragraph, because we have provided evidence about this in ETAPlaT protocol paper.
8. Page 6 Efficacy analyses of secondary outcomes, 2nd paragraph: We removed this section.
9. Figure 1: ****Here we follow standards which define the boxplot. The specific definitions are now added to the SAP.
10. General comments: We follow your advice already given regarding page 4 and 5.
11. CONSORT Flowchart: Good point: Will be added to the SAP
12. Adherence: We added a section adherence to the revised SAP.

# Peer review: Referee response for version 1

**DOI:** 10.21956/wellcomeopenres.10886.r21539

**Published:** 2017-04-04

**Authors:** Christel Weiß

**Affiliations:** 1 Department of Biometry and Statistics, Medical Faculty Mannheim, University of Heidelberg, Mannheim, Germany

The sample size has been assessed assuming a 1-sided 2 sample t test which will be performed in order to compare the changes. This should be mentioned (1-sided). Furthermore, patients will be included in the primary efficacy analysis if only the baseline measurement is available. If a rather big number of patients will not have the 30-minute-measurement then the sample size off 88 per group may be too small.

I have read this submission. I believe that I have an appropriate level of expertise to confirm that it is of an acceptable scientific standard.

# Response

**Published:** 2017-05-29

**Authors:** Kastriot Dallaku

**Affiliations:** Hospital of Ludwig Maximilian University of Munich, Germany

Thank you for your comments. We will impute missing values of baseline measurements and modified this issue in our revised SAP. It was clear from the performance in the single center that the problem of missing baseline measurements will not be severe.

# Peer review: Referee response for version 1

**DOI:** 10.21956/wellcomeopenres.10886.r20785

**Published:** 2017-03-31

**Authors:** Stephen J. Senn

**Affiliations:** 1 Competence Centre for Methodology and Statistics, Luxembourg Institute of Health, Strassen, Luxembourg

I have approved this report with reservations because my opinion is that the plan is generally sound and prepared in sufficient detail. Nevertheless, I consider that there is one aspect that is puzzling and might be improved.

 As part of the sample size determination, the authors calculate the variance of the change from baseline measure of ETP This might be fair enough for the purpose of sample size determination. However, it is well known that this is an inefficient use of baselines and that one can do better by including the baseline as a covariate ^1^. The section headed **Efficacy analyses of primary outcome** states:

*In analysis of the primary outcome(TGA– ETP), the TXA group will be compared with the placebo group, based on analysis of covariance (ANCOVA) which adjusts for the length of time between two measurements (30±15 minutes) of the baseline and follow-up TGA - ETP value*

 This does not, therefore, appear to adjust for the baseline value (apart for the crude and inefficient adjustment through subtraction). On the other hand, there is an adjustment for the length of time between the two measurements. This implies that this length of time is itself considered to be predictive. That is to say it is supposed that two women given identical treatment would be expected to have different values of TGA according to the length of time it takes to measure TGA. This may well be reasonable (if some secular trend in TGA is expected) but in that case I would have expected some discussion of the point.

 In short, I consider that the plan shows evidence of careful thought on the part of the authors but still leaves room for some doubt as regards the analysis. I must thus conclude that either the analysis proposed is not optimal or that the authors have failed to provide sufficient clarity to entirely convince at least one reader of its appropriateness.

I have read this submission. I believe that I have an appropriate level of expertise to confirm that it is of an acceptable scientific standard, however I have significant reservations, as outlined above.

## References

[1] SennS: An unreasonable prejudice against modelling?. *Pharmaceutical Statistics*.2005;4(2) : 10.1002/pst.169 87-89 10.1002/pst.169

# Response

**Published:** 2017-05-29

**Authors:** Kastriot Dallaku

**Affiliations:** Hospital of Ludwig Maximilian University of Munich, Germany

Many thanks for this comment. We will add the baseline adjustment to the linear model used to quantify treatment effects.

# Peer review: Referee response for version 1

**DOI:** 10.21956/wellcomeopenres.10886.r18944

**Published:** 2017-02-06

**Authors:** Anne-Mette Hvas

**Affiliations:** 1 Department of Clinical Biochemistry, Centre for Haemophilia and Thrombosis, Aarhus University Hospital, Aarhus, Denmark

The title is appropriate.

 The abstract provides an adequate summary of the manuscript.

 The presented study is scientifically sound; The study is well-designed, it seems well conducted and the laboratory methods used to investigate the research questions are relevant. However, the statement on whether a possible effect of TXA is due to inhibition of plasmin cannot be investigated (the authors don't investigate the mechanisms of the TXA-effect), so that is a theoretical deduction. The statistical approach is clearly and comprehensively stated.

I have read this submission. I believe that I have an appropriate level of expertise to confirm that it is of an acceptable scientific standard.

# Response

**Published:** 2017-05-29

**Authors:** Kastriot Dallaku

**Affiliations:** Hospital of Ludwig Maximilian University of Munich, Germany

Thanks for this comment and the clear formulation of the limitation given by our approach. This comment will be relevant when discussing the results of the trial.
